# Maize Centromere Structure and Evolution: Sequence Analysis of Centromeres 2 and 5 Reveals Dynamic Loci Shaped Primarily by Retrotransposons

**DOI:** 10.1371/journal.pgen.1000743

**Published:** 2009-11-20

**Authors:** Thomas K. Wolfgruber, Anupma Sharma, Kevin L. Schneider, Patrice S. Albert, Dal-Hoe Koo, Jinghua Shi, Zhi Gao, Fangpu Han, Hyeran Lee, Ronghui Xu, Jamie Allison, James A. Birchler, Jiming Jiang, R. Kelly Dawe, Gernot G. Presting

**Affiliations:** 1 Molecular Biosciences and Bioengineering, University of Hawaii, Honolulu, Hawaii, United States of America; 2 Division of Biological Sciences, University of Missouri, Columbia, Missouri, United States of America; 3 Department of Horticulture, University of Wisconsin–Madison, Madison, Wisconsin, United States of America; 4 Department of Genetics, University of Georgia, Athens, Georgia, United States of America; Fred Hutchinson Cancer Research Center, United States of America

## Abstract

We describe a comprehensive and general approach for mapping centromeres and present a detailed characterization of two maize centromeres. Centromeres are difficult to map and analyze because they consist primarily of repetitive DNA sequences, which in maize are the tandem satellite repeat CentC and interspersed centromeric retrotransposons of maize (CRM). Centromeres are defined epigenetically by the centromeric histone H3 variant, CENH3. Using novel markers derived from centromere repeats, we have mapped all ten centromeres onto the physical and genetic maps of maize. We were able to completely traverse centromeres 2 and 5, confirm physical maps by fluorescence *in situ* hybridization (FISH), and delineate their functional regions by chromatin immunoprecipitation (ChIP) with anti-CENH3 antibody followed by pyrosequencing. These two centromeres differ substantially in size, apparent CENH3 density, and arrangement of centromeric repeats; and they are larger than the rice centromeres characterized to date. Furthermore, centromere 5 consists of two distinct CENH3 domains that are separated by several megabases. Succession of centromere repeat classes is evidenced by the fact that elements belonging to the recently active recombinant subgroups of CRM1 colonize the present day centromeres, while elements of the ancestral subgroups are also found in the flanking regions. Using abundant CRM and non-CRM retrotransposons that inserted in and near these two centromeres to create a historical record of centromere location, we show that maize centromeres are fluid genomic regions whose borders are heavily influenced by the interplay of retrotransposons and epigenetic marks. Furthermore, we propose that CRMs may be involved in removal of centromeric DNA (specifically CentC), invasion of centromeres by non-CRM retrotransposons, and local repositioning of the CENH3.

## Author Summary

Centromeres tend to be the last regions to be assembled in genome projects, as their mapping is hampered by their characteristically high repeat DNA content and lack of genetic recombination. Using unique markers derived from these repeat-rich regions, we were able to generate and annotate physical maps of two maize centromeres. Functional centromeres are defined not so much by their primary DNA sequence as by the presence of CENH3, a special histone that replaces canonical histone H3 in centromeric nucleosomes. Little is known about how deposition of CENH3 is regulated, or about the interplay between centromeric repeats and CENH3. By graphing the density of CENH3 nucleosomes onto the physical map, we delineated the functional centromeres in today's maize genome. We then used the large number of LTR retrotransposon insertions, for which the corn genome is well known, as “archeological evidence” to reconstruct the historic centromere boundaries. This was possible because i) some retrotransposon families of maize (CRM) appear to possess a unique ability to preferentially target centromeres during integration and ii) insertion times of individual retrotransposons can be calculated. Here we show that the centromere boundaries in maize have changed over time and are heavily influenced by centromeric and non-centromeric repeats.

## Introduction

LTR retrotransposons are useful tools for understanding genome evolution because of their target site specificity and our ability to estimate their insertion times based on sequence divergence of their LTRs [1]. Retrotransposons account for >75% of the maize genome sequence [2] and are responsible for much of the genome expansion that has taken place since the allotetraploidization event that gave rise to present day maize [3],[4].

Centromeric retrotransposons (CR) were initially discovered as centromere-specific sequences in the grasses [5],[6]. The CRs of maize (CRM) and rice (CRR) belong to distinct subfamilies [7]–[9], which have been grouped most recently into four orthologous subfamilies [9]. One of these subfamilies, CRM1, has proliferated extensively in the past 3–4 million years by generating at least 5 recombinant subgroups from two parental variants thought to have been combined in the maize genome during allotetraploidization [10]. No full-length element of the CRM1-orthologous rice subfamily (CRR3) is found in the *O. sativa* ssp. *japonica* genome, raising doubt as to whether CR elements in general, and CRM1 in particular, are required for centromere function. With the exception of members of the recently discovered CRM4 subfamily, all known CRM elements localize almost exclusively to centromere regions as determined by fluorescence *in situ* hybridization [11],[12], and physical mapping [2]. The mechanism of centromere localization is as yet unknown.

Like the centromeres of most eukaryotes, plant centromeres also contain tandem satellite repeats [7], [13]–[16]. Tandemly arranged CentC repeats (monomer length ≈156 nt) and interspersed CRM are the major DNA components of maize centromeres [7],[15],[17], but their role in centromere function is unclear. The satellite sequences of corn and rice, which diverged from a common ancestor approximately 50 MYA [18],[19], exhibit regions of high sequence similarity [20] and are clearly homologous.

Functional centromeres of all eukaryotes examined to date are marked epigenetically by a centromeric histone H3 (CENH3), which replaces the canonical histone H3 in centromeric nucleosomes [21]. A key question in centromere biology is how deposition of CENH3 in centromere regions is controlled. Chromatin immunoprecipitation (ChIP) experiments with anti-CENH3 antibodies is an effective method for isolating centromeric chromatin [15], and has been used previously to perform a comparative study of rice centromeric satellite sequences [20] and to precisely delineate the borders of several rice centromeres [22].

Excellent cytogenetic and genetic resources, including oat-maize addition lines that carry a single maize chromosome in an oat background [23], together with the recently published reference genome [2] of the maize inbred B73 (ZmB73v1), make maize a good model for studying centromeres. Here we present the physical maps of maize centromeres 2 and 5, on which the functional centromeres have been precisely delineated using anti-CENH3 ChIP sequences. The highly active retrotransposon population of maize provides a detailed record of centromere evolution that is unattainable from smaller genomes with fewer or less active retrotransposons.

## Results

### Genetic map positions of all ten maize centromeres

Two methods were employed to identify molecular markers that can be used to genetically map maize centromeres, which consist largely of repetitive sequences. We used both the repeat junction method [24] and transposon display [25],[26] with CRM2 to generate a total of 54 centromere-derived polymorphic markers (Tables S1, S2) that could be placed onto the maize genetic map using a mapping population [27] derived from inbreds B73 x Mo17. This simultaneously anchored centromeric BACs to their respective chromosomes (Tables S1, S2) and provided the genetic map positions for all ten centromeres (Table 1).

**Table 1:** Genetic and physical map locations of all 10 maize centromeres.

Chromosome	Centromere Marker	Chromosome Position (cM)	Genetic Marker Interval	Estimated Physical Map Position Based on Genetic Markers	Map Position of Functional Centromeres
1	Cent1	439.3	csu1138-umc1076	121.0 Mb–133.1 Mb	133.3 Mb–133.9 Mb
2	Cent2	344.8	umc1581-zpu1	92.2 Mb–101.6 Mb	89.3 Mb–91.1 Mb
3	Cent3	254.0	AY111333-AY110151	87.4 Mb–89.4 Mb	94.6 Mb–95.4 Mb
4	Cent4	298.9	umc1791-bnlg1755	71.8 Mb–93.0 Mb	104.2 Mb–105.0 Mb
5	Cent5	313.3	umc1283-umc1591	94.8 Mb–118.4 Mb	101.6 Mb–104.8 Mb 107.6 Mb–108.6 Mb
6	Cent6	98.0	uck1-umc1444	31.8 Mb–32.7 Mb	49.8 Mb–50.4 Mb
7	Cent7	184.1	umc1879-umc1409	44.9 Mb–54.1 Mb	55.3 Mb–55.7 Mb
8	Cent8	207.9	umc1904-rps28	55.8 Mb–80.7 Mb	45.9 Mb–48.0 Mb
9	Cent9	224.7	gpm46-pep1	34.1 Mb–34.2 Mb	68.6 Mb–69.2 Mb
10	Cent10	191.4	bnlg1716-umc2067	37.1 Mb–46.0 Mb	59.3 Mb–60.7 Mb

Functional centromeres were defined as the region on each chromosome where the moving average of MUMmer ChIP reads per three 100 kb windows was ≥20, except for chromosomes 1, 6, 7, and 9, where BLAST and a moving average of ≥30 (≥15 for chromosome 6) per three 100 kb windows was used to define the centromere. Physical map coordinates are based on reference chromosomes of ZmB73v1 [2].

### Physical maps of centromeres 2 and 5

Using BAC sequence data from the Maize Genome Project [2], fingerprinted contigs (FPC) data from the Arizona Genomics Institute [28] (ftp://ftp:agiftpguest@ftp.genome.arizona.edu/pub/fpc/maize/), and the centromeric markers described above, we were able to construct physical maps traversing the entire centromere on chromosomes 2 and 5. Our BAC-based physical maps for these two centromeres are largely in agreement with the reference chromosomes presented of the B73 reference genome ZmB73v1 [2], thus reference chromosome coordinates are provided for the features we describe here. The main difference between these maps is the closure of a gap on centromere 5 (position 105,074,634) using the CentC-rich singleton BAC ZMMBBb0271K07, which has not yet been incorporated into reference chromosome 5. Even excluding this BAC, the CentC content of centromere 5 is about 3 times higher than that of centromere 2.

**Figure 1:**
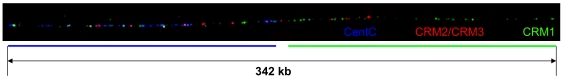
Fiber FISH map of a 342 kb region within the approximately 7 Mb B73 centromere 5. An oat-maize addition line for B73 chromosome 5 shows a predominantly CentC-containing region with interspersed CRMs that is flanked by a CRM1-rich region. CentC  =  blue, CRM1  =  green, CRM2/CRM3  =  red.

Centromeres 2 and 5 of B73 contain very little CentC as compared to the other eight centromeres [29]. Fiber FISH using CRM and CentC probes on B73 oat-maize addition lines carrying a single maize chromosome (2 or 5), indicate that CentC repeats are confined to a few small blocks interspersed with CRM in both of these centromeres (Figure S1 and Figure 1, respectively). Measurements of the stretched chromosomes show that these CentC blocks of centromeres 2 and 5 span approximately 196 kb (Figure S1) and 192 kb (Figure 1), respectively. The physical map of centromere 2 (for a graphical representation of the entire region please see [30]) is in good agreement with the FISH data as it contains a number of short CentC repeat clusters totaling about 31 kb and ranging in size from about 1 kb to 15 kb. These clusters span an approximately 130 kb region near the center of the functional centromere [30], which is close to the fiber FISH estimate. The difference between the two maps is most likely due to the fact that the physical map still contains numerous gaps and consists of relatively small sequence fragments of unknown order and orientation. Similarly, the physical map of centromere 5 shows one major region of CentC spanning 246 kb [30], and a repeat arrangement similar to that shown by fiber FISH (Figure 1), i.e. distinct CentC- and CRM1-rich regions.

CRM1 and CRM2 constitute the majority of centromeric repeats (CRM and CentC) present in these two centromeres (94% and 80% for centromeres 2 and 5, respectively), but the ratio of CRM1 to CRM2 in centromere 5 is about double that of centromere 2 (Table 2).

**Table 2:** CRM and CentC content of centromere regions 2 and 5.

Chromosome	2	5
Reference chromosome coordinates	87.1–93.5 Mb	99.3–110.1 Mb
Mapped MUMmer Reads	1,291	1,843
Mapped BLAST Reads	2,200	3,350
CRM1 (nt)	296,917	490,825
CRM2 (nt)	354,264	282,270
CRM3 (nt)	0	42,194
CRM4 (nt)	11,734	56,716
Ratio of CRM1/CRM2	0.84	1.74
CentC (nt)	31,550	89,593
Approx. Number of CentC Monomers	202	574

### Delineation of functional centromeres 2 and 5 by chromatin immunoprecipitation

**Figure 2:**
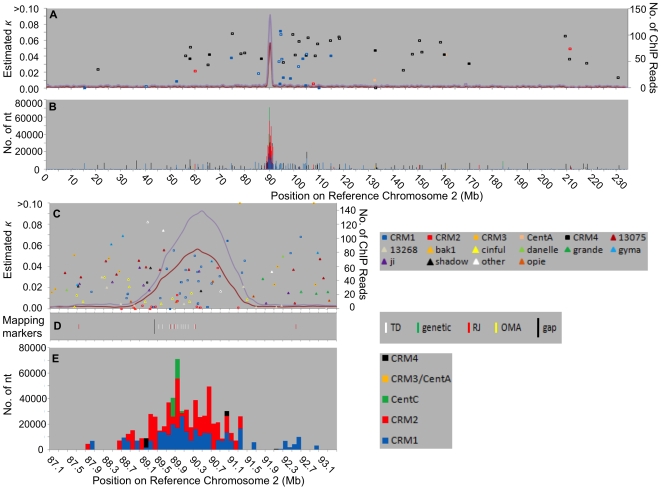
Fine-scale physical maps of centromere 2. (A,B) Chromosomal views. (A) Moving average of 9 windows of the number of sequence reads mapped per 100 kb window using MUMmer (red line) or BLAST (purple line) [2]. Colored boxes denote single CRM elements whose insertion was dated using the method of San Miguel et al. [1]. Only elements that have inserted outside of the functional centromere are shown. Filled squares  =  full-length elements, empty squares  =  fragmented elements. κ  =  estimated number of nucleotide substitutions per site. (B) centromeric repeats CRM1, CRM2, CRM3, CRM4 and CentC mapped onto the reference chromosomes using competitive BLAST and graphed as number of nucleotides per 100 kb window. (C–E) Close-up of centromere region. The functional centromere plus approximately 2.3 Mb of pericentromeric region are shown. (C) CENH3 data same as (A). Retroelements include CRMs not pictured in (A) and non-CRM elements (triangles - details in Table S6); filled symbols  =  full-length elements, empty symbols  =  fragmented elements. Only two bak1 elements have *κ*>0.1 and are located at 91,278,432 (*κ* = 0.24) and 92,902,773 (*κ* = 0.16) and for space reasons are drawn at κ = 0.1. (D) Genetic and molecular markers used to anchor this region to chromosome 2 – see Table S7 for details. Large vertical bar denotes contig gap in reference chromosome. (E) Centromeric repeats as in (B).

**Figure 3:**
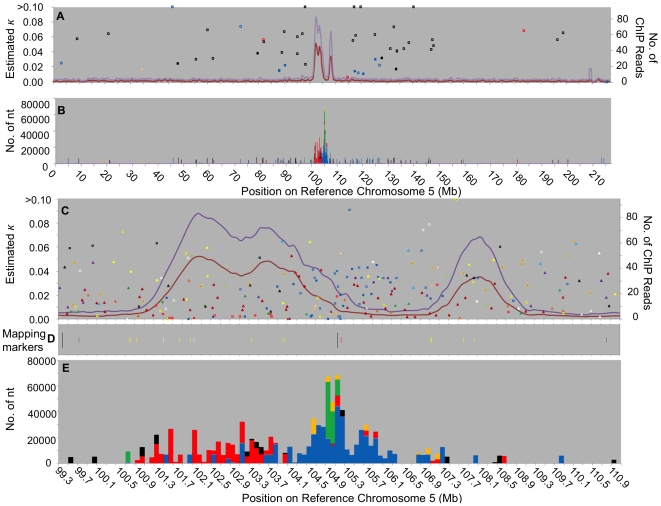
Fine-scale physical maps of centromere 5. Panels and legend as in Figure 2. Elements with κ>0.1 include: (A,B) a CRM1 at 45,864,292 (*κ* = 0.125), CRM4s at 96,757,409 (*κ* = 0.101), 115,640,763 (*κ* = 0.14), 118,276,888 (*κ* = 0.18), and 138,633,510 (*κ* = 0.172), (C,D) cinful at 107,583,697 (*κ* = 0.11) and 107,592,411 (*κ* = 0.11).

We used ChIP with anti-CENH3 antibody followed by pyrosequencing to generate 149,756 mostly centromere-derived DNA sequences of maize inbred B73 with an average high quality read length of 165 nt and totaling 24,729,204 nt. The availability of high quality sequence covering all regions of the maize genome represented in FPC contigs of the AGI physical map [31] allowed us to map the immunoprecipitated sequences onto the physical map using MUMmer and BLAST [2], thereby delineating the functional centromeres on all ten reference chromosomes (Figure 2A, Figure 3A, Figure S2). MUMmer, which was used to map reads to the genome at 100% identity over 100% of the read length, allowed us to anchor 44,897 ChIP sequences. Of the remaining sequences, 59,913 were mapped by BLAST using cutoffs of 96% identity over 96% of the ChIP read length. The reads that could not be mapped using these BLAST parameters likely represent centromeric regions that are missing in the ZmB73v1 reference genome assembly, which contains only an estimated 54% of the genome's total CentC content [2]. The BLAST and MUMmer reads are graphed as moving averages onto the reference chromosomes – both peak at the regions of highest centromere repeat density on all chromosomes (Figure 2A and 2B, Figure 3A and 3B, Figure S2). On chromosome 2, the arms exhibit a background signal of about 2.1 reads per 100 kb window, which is approximately 30 times lower than the read count of the centromeric peak (Table S3). This background signal is likely due to co-purification of non-centromeric chromatin during the initial chromatin immunoprecipitation with anti-CENH3 antibody, as reflected by the small amount of background signal visible on the chromosome arms in FISH performed with the ChIP fraction (Figure S3). The major FISH signal corresponds to the ten centromeres, indicating significant enrichment of the CENH3 chromatin fraction. Several smaller peaks formed by reads with less than 100% identity are found in euchromatic regions of several chromosomes and correspond to knob repeats or plastid sequences. Most chromosomes contain a single CENH3 peak that correlates with a high centromere repeat density (Figure S2). For the chromosomes containing more than one centromere peak, we were able to identify the correct centromere position using the genetically mapped centromeric markers (Tables S1, S2).

About 13.6% (20,441) of the ChIP reads were not mapped because they did not meet the minimum BLAST length or identity requirements. Many of these are likely to be centromeric as they were classified as CRM (1,936) or CentC (766) based on cross_match (http://phrap.org) of 100% of the read. Another 24,505 reads (7,330 CRM and 758 CentC) mapped to multiple regions with identical bitscores and are also not graphed on the reference chromosomes. This illustrates the problem that, although we were able to reliably classify ChIP reads as CRM or CentC, mapping a read to a single location in the genome is possible only for those reads containing a unique SNP. As a result, it is difficult to determine if any given CRM element is associated with CENH3 nucleosomes, especially if it has inserted recently.

The functional centromere 2 is defined by a single CENH3 binding domain of 1.8 Mb (Figure 2A and 2C; Table S3). This is relatively large compared to the size of the four best-sequenced rice centromeres, which span 420–820 kb [22] but move chromosomes that are on average about five times smaller than the maize chromosomes (41 Mb vs 200 Mb). On centromere 5 the mapped ChIP reads reveal two distinct CENH3-containing regions of sizes 3.2 Mb (“L”  =  left) and 1.0 Mb (“R”  =  right), separated by a circa 2.8 Mb interstitial (“I”) region exhibiting near background ChIP levels and discernable even at the whole chromosome level (Figure 3A and 3C; Table S4). Both of these blocks are anchored to centromere 5 by a number of markers, including repeat junction, transposon display, oat-maize addition line and genetic markers (Figure 3D, Table S7, Table S8), which provide a high confidence level of the accuracy of the physical map. Thus we are confident of the location of the “R” region even though this CENH3-rich region is virtually devoid of centromeric repeats, making it difficult to detect and verify by FISH. Note that a complete physical map traversing an entire centromere is required to detect multiple CENH3 domains, which may exist in some of the other eight centromeres for which the physical maps are not yet completely assembled.

As detailed above, the “L” and “R” blocks of centromere 5 together are 2.3 times larger than the entire functional centromere 2. Remarkably, the smaller centromere 2 CENH3 region contains a higher density of CENH3 ChIP reads, such that the total number of reads mapped to each centromere is 1,130 for centromere 2 (1.8 Mb) and 1,562 (1,247 in “L” plus 315 in “R”) for centromere 5 (4.2 Mb). Note that the number of reads mapped to each centromere is only an approximate and indirect estimation of the number of CENH3 nucleosomes, and that this number is heavily influenced by the number of unique targets available in each region to which the reads can be mapped. Nevertheless, it appears as though the difference in centromere size is compensated somewhat by the density of CENH3 nucleosomes, measured indirectly as 628 ChIP reads/Mb for centromere 2, and 390 and 315 ChIP reads/Mb for centromere 5 “L” and 5 “R”, respectively.

### Centromeric repeats are distributed differently in centromeres 2 and 5

Figure 2B and 2E and Figure 3B and 3E illustrate the centromeric repeat content (CRM and CentC) of centromeres 2 and 5 in non-overlapping 100 kb segments. These repeats reach local maxima of up to 91% per 100 kb window (chromosome 9 in Table S5). For centromere 2, repeat content of these windows correlates well with the CENH3 content (Figure 2A and 2C). The central CentC region is flanked on both sides by CRM1 and CRM2 elements. CRM1 sequence is present at slightly lower levels than CRM2 throughout the functional centromere (Table S3; Figure 2E) and is found in small amounts in the flanking regions up to 2 Mb away.

In addition to consisting of two distinct CENH3 domains, centromere 5 differs from centromere 2 in that the centromeric repeats are not distributed evenly. A small amount (17 kb) of CentC lies outside of the functional centromere at 100.7 Mb. The larger CENH3 block (“L”) contains predominantly CRM2 and a smaller amount of CRM1 (Table S4; Figure 3E). Unlike centromere 2, the largest block of CentC in centromere 5 lies at the right edge of this block (105 Mb), the “L”/”I” border. A number of CRM elements have inserted into this CentC cluster, which is flanked on both sides by large amounts of CRM1. This has resulted in a skewed CRM1/CRM2 distribution on centromere 5, with a CRM2-rich region in the left half of “L” and a CRM1-rich region at the “L”/”I” border that extends about halfway into “L” on one side, and into the CENH3-poor interstitial region on the other. The second, smaller CENH3 block of centromere 5 (“R”) contains very little centromeric repeat.

### CRM elements localize predominantly, but not exclusively, to active centromeres

As was expected from published FISH experiments, CRM elements belonging to the CRM1, CRM2 and CRM3 subfamilies are localized primarily to centromeres (Figure 2B, Figure 3B, Figure S2). However, the physical maps do reveal small amounts of CRM1 and CRM2 sequences on most chromosome arms that would be difficult to detect by FISH. In some cases, these sequences represent a single element that may have inserted aberrantly. For example, element CRM1_18 near the telomere of 5L (position 213,233,223), which encodes an otherwise functional polyprotein, contains a mutation in the conserved chromodomain that might have impaired target-specific integration of this element. Its 5′ and 3′ LTRs are identical, indicating that this element inserted within the past 150,000 years. Other CRMs may have been translocated to chromosome arms from an initially centromeric position as part of another retrotransposon or helitron, though we have found no evidence for this to date. While mindful of these exceptions, we postulate that CRM elements predominantly target functional centromeres, and that the CRM insertions dated by the method of San Miguel et al. [1] therefore represent a historical record of centromere location over evolutionary time. This is supported by the fact that virtually all CRM elements with identical LTRs (*κ* = 0) are located within the current CENH3 region as delineated by the ChIP reads.

### Retrotransposons are major features of centromeres 2 and 5

**Figure 4:**
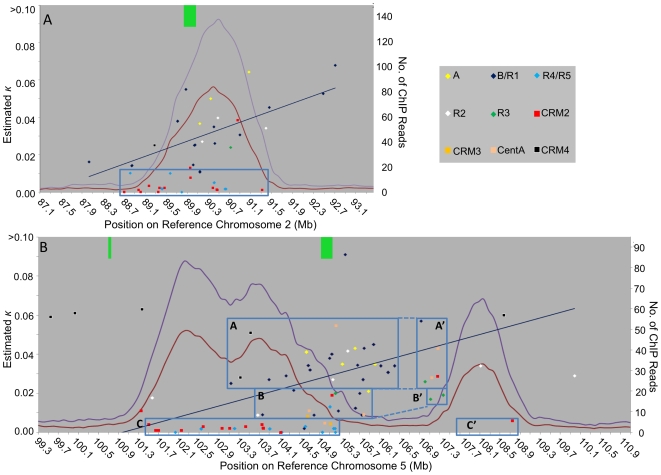
Successive centromere invasion by different CRM1 recombinant subgroups and CRM2 document centromere location over time and the progressive split of centromere 5. CRM elements are graphed by chromosome coordinate and insertion time (κ) for (A) centromere 2 and (B) centromere 5. Note that the more recent insertions represented by the more recently derived recombinant CRM1 elements R4 and R5 are tightly associated with the present-day CENH3 region. Older elements indicate CENH3 location in the past. Linear regression lines were calculated for all elements of the ancestral CRM1 B/R1 subfamily and document the shift of the centromeres over time. Boxes denote approximate centromere positions at different times based on CRM elements for which the time of insertion could be calculated, except for box C′, the left and right borders of which are based on the CENH3 data and the sole CRM element, respectively. Boxes illustrate the gradual increase in size of “I” over time. Green bars denote approximate positions of CentC clusters.

We were able to date the insertion time of a large number of retroelements that inserted in or near the functional centromeres 2 (128 elements) and 5 (246 elements). The locations and dates of these insertions provide a powerful tool for elucidating centromere dynamics over evolutionary time. In general, recently (*κ* ≤0.01) inserted CRM elements are located within the CENH3 regions, while non-CRM retrotransposons that inserted during the same period tend to be present in higher numbers outside of the centromeres (Figure 2C, Figure 3C). In accordance with the CRM1 element evolution described by Sharma et al. [10], the youngest CRM1 element insertions that are located in the centromere 2 CENH3 region and centromere 5 “L” region consist exclusively of the most recently formed recombinants R4 and R5, while the older CRM1 elements lie closer to the border or outside of the current CENH3 region on both centromeres and belong to the older recombinant (R3, R2, R1) or parental (A and B) types (Figure 4). The fact that recent CRM1 insertions are located almost exclusively in the current CENH3-containing region while older CRM1 elements are located both within that region as well as in nearby chromatin, suggests that the CENH3-containing region, and thus the functional centromere, can shift locally over time.

### CRM1 elements do not appear to cause formation of a functional centromere

The centromere 5 picture is complex: a large number of non-CRM retrotransposons appear to have inserted into both the CENH3-rich and the surrounding regions. Also, a large number of CRM1 elements have inserted near the major CentC cluster on the border of the “L” and “I” regions (Figure 3E, Figure 4B). Recent CRM insertions are located exclusively within the left functional domain, while the CRM1 elements in the “I” region have inserted at progressively older times the farther they are located from the CentC cluster (see trend lines in Figure 4B). Conversely, the youngest non-CRM elements have inserted predominantly in the interstitial and pericentromeric regions. In other words, within the functional domain “L” it is the CRM elements that have inserted after the non-CRM elements, whereas in the interstitial region the CRM1 elements have inserted before the other types of elements.

Finally, individual CRM1 elements of similar (old) age vary in the number of ChIP reads mapped to each element in accordance with their chromosomal location: those elements located within the “L” or “R” block exhibit a higher number of ChIP reads than elements of the same age located in the interstitial region or on the long arm of chromosome 5 (Figure S4). Taken together these observations indicate that CRM1 elements do not cause the formation of functional centromere chromatin but simply possess an extremely efficient mechanism that targets their insertion into CENH3-containing chromatin.

### FISH confirms that CRM2 is associated with the kinetochore

**Figure 5:**
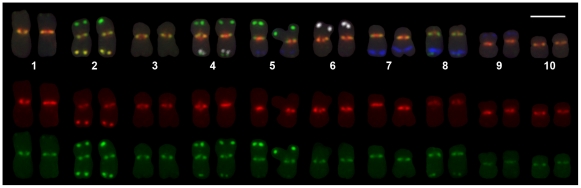
Karyotype of maize inbred line B73 illustrating CRM1 and CRM2 distribution. CRM1 was labeled with Texas Red and CRM2 with AlexaFlour 488 (green). Other features that permit the classification of each chromosome are 180 bp knob repeat labeled with Cascade Blue, subtelomeric probe 4-12-1 and 5S ribosomal RNA labeled with AlexaFluor 488, 5S rDNA with Texas Red (to produce a yellow composite) and the TR1 knob repeat labeled with Cy5 (pseudocolored white). The alignment of red and green labelings of the 5S cluster on chromosome 2 assures the relative alignment of CRM1 and CRM2 in the centromeric regions. Note that centromeres 2 and 8 contain relatively high amount of CRM2 relative to CRM1. The reverse is true for centromere 9. The merged image is at the top. The Texas Red signal is shown in the middle panel that includes CRM1 at the primary constriction and the lower panel green image includes the CRM2 signal at the primary constriction. Chromosomes are representative of multiple metaphase cells each observed from root tip biological replicates. Scale bar  = 5 µm.

**Figure 6:**
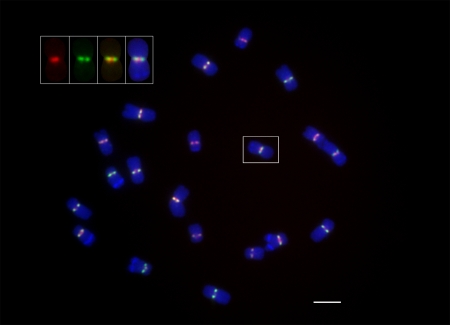
Relative positioning of CRM1 and CRM2 on somatic metaphase chromosomes. A somatic root tip chromosome spread of B73 is presented and labeled with CRM1 and CRM2 as described in Figure 5. As determined from the karyotyping features shown in Figure 5, the presence of a smaller interstitial knob on the long arm identifies the boxed chromosome as 5. The inset to the upper left illustrates the different channels from left to right, CRM1, CRM2, CRM1+CRM2 and the chromosome composite. As is generally the case with the chromosomes in the spread, CRM1 label has a more internal positioning than CRM2, which lies to the exterior of the chromosome opposed to the sites of sister chromatid cohesion although there is also obvious overlap. The metaphase spread is representative of multiple metaphase cells each observed from root tip biological replicates. Scale bar  = 5 µm.

A combination of four repetitive element probes allows identification of all B73 chromosomes in FISH experiments (Figure 5). Novel CRM1- and CRM2-specific probes were used to assess the distribution and arrangement of these two CRM subfamilies on metaphase chromosomes. While all centromeres contain visible amounts of both CRM1 and CRM2, centromere 9 appears to contain relatively little CRM2, which may explain our inability to derive CRM2 transposon display markers for this centromere. Figure 5 and Figure 6 further demonstrate that CRM1 and CRM2 elements are distributed in overlapping but somewhat distinct positions on the metaphase chromosomes. In general, CRM2 appears to localize to the exterior centromere face of chromosome 5 and other chromosomes, while CRM1 appears to be more prominent in the sister chromatid cohesion region, but with overlap clearly observed between the two probes (Figure 5, Figure 6).

Thus the FISH data are consistent with the CENH3 distribution inferred from our ChIP mapping, and we now have three lines of evidence suggesting that CRM2 is more closely associated with CENH3 than CRM1. First, at the genomic scale, enrichment of CRM2 in CENH3 ChIP is about three times higher than CRM1 enrichment [2]. Second, the CENH3-containing regions of both centromeres 2 and 5 contain more CRM2 than CRM1 (Tables S3, S4). Finally, FISH experiments with subfamily-specific FISH probes for CRM1 and CRM2 indicate that CRM2 appears to be preferentially localized to the exterior face of the centromere, whereas CRM1 localizes predominantly to the inter-chromatid region.

However, although this tighter association of CRM2 with CENH3 is true when considering the distribution of CRM1 and CRM2 on a genomic scale, the physical maps of centromeres 2 and 5 clearly illustrate that the young subgroups of CRM1 (R4 and R5) mirror the location of CRM2 (Figure 4), and that the likelihood of an element being located within the CENH3 region appears to be a function of the time at which a given CRM element has inserted rather than the subfamily to which it belongs.

## Discussion

### Centromere mapping

We used two novel but generally applicable methods for deriving centromere markers that were critical for anchoring the centromeres to both the physical and genetic maps. First, we used a modified transposon display method [25],[26] to screen a large number of potential centromeric markers for polymorphisms between the two parents of the IBM mapping population. In essence this is a centromere-specific AFLP screen that utilized the LTR of the abundant centromere-specific CRM2 retrotransposon as one of the priming sites. Polymorphic AFLP bands were mapped onto the IBM population and subsequently cloned, sequenced, and mapped onto the BAC sequences provided by the Maize Genome Sequencing Consortium [2].

The second method is based on the use of PCR primers derived from repeat junctions identified on centromeric, i.e. CentC- or CRM-containing, BAC clones [24]. JunctionViewer software [30] was used to identify repeat junctions located within 2.5 kb of each other (e.g. resulting from nested insertions). Primers were subsequently designed on these junctions and tested for polymorphism between the mapping parents. Finally, polymorphic markers were mapped onto the genetic map using the IBM population. In contrast to the transposon display method, the junction method utilizes junctions between all types of centromeric repeats, and thus provided a complementary marker set, particularly for centromeres containing relatively little CRM2 (e.g. centromere 9). However, the large number of potential markers that had to be screened individually, as well as the very precise PCR reaction conditions required to produce differential amplification in the two mapping parents, made this a very labor-intensive method for finding centromere-specific markers. Both repeat junction and CRM2 display markers are dominant markers that were effective in anchoring centromeres to genetic and physical maps.

The identification of novel centromeric markers using the repeat junction and transposon display methods, in combination with anti-CENH3 ChIP followed by pyrosequencing allowed us to precisely delineate the edges of the functional centromeres on all chromosomes. On most chromosomes the CENH3 nucleosomes map to a single region, but on several chromosomes additional peaks are observed. In some cases the additional peaks are caused by underlying knob repeats. However, unlike what is observed at centromere peaks, few if any reads that map to these knob repeats are 100% identical to their target. Therefore we believe that these peaks are generated by reads that originate from the estimated >90% of knob repeats that are absent from the maize reference genome ZmB73v1 [2] mapping to the best heterologous location available on the reference chromosomes. However, we cannot completely exclude the possibility that some knob repeats are associated with CENH3 in the other eight centromeres that have not yet been completely assembled.

### Maize centromeres are sites of active genome rearrangement

We were able to construct physical maps that traverse the entire B73 centromere region for two chromosomes, allowing us for the first time to analyze the repeat content and arrangement in the context of a complete maize centromere. These two centromeres are unusual in that they contain small amounts of CentC satellite as confirmed by FISH experiments [29]. However, other maize inbreds do contain large amounts of CentC in centromeres 2 (B37, KYS, W22) and 5 (K10, Stock6) [29]. The presence of the related CentO satellite in all rice centromeres, the fact that the related *Tripsacum* has high levels of CentC at all centromeres but much lower and highly variable levels of CRM on different centromeres [12], and the fact that rice contains few CR elements compared to maize [9] lead us to believe that the CentC satellite represents an ancient form of centromere repeat and that the low CentC-containing centromeres 2 and 5 of B73 represent relatively recent changes.

The restriction of recent CRM1 insertions (*κ* ≤ 0.01 = <750,000 years ago) on centromeres 2 and 5 to the current CENH3 domains indicates that these elements are equipped with an effective targeting mechanism that directs the majority of these elements into active centromeres. Chromatin components are thought to play a role in directing the yeast Ty elements to their chromosomal targets [32]. Furthermore, Lamb et al. [33] discovered that maize retrotransposon families are enriched in distinct patterns on maize chromosomes and noticed a correlation between insertion patterns of opie and prem2/ji with the modified histone H3K4me2. Finally, the chromodomain of the fungal chromovirus MAGGY integrase protein has been shown to interact with a certain methylated histone H3 variant and direct integration of heterologous retroelements to chromosomal regions containing these variants [34]. Thus, although the exact targeting mechanism for CRM elements remains to be determined, CENH3 [8] or centromere-specific histone H3 methylation variants [35],[36] represent plausible candidates for directing these elements to centromeres.

Regardless of the targeting mechanism, CRM elements provide a record of the centromere location over evolutionary time and can be used to recreate centromere evolution. This is illustrated particularly well by the major CRM1 cluster of centromere 5 flanking the “L”/”I” border region and the major CentC cluster (Figure 3C): for the CRM1 (as well as the much less numerous non-autonomous CentA) elements located in the interstitial region, there is a direct correlation between the element's distance from the “L”/”I” border and its insertion time, presumably because they were pushed away from the active centromere region by subsequent CRM1 insertions into the CENH3 region when it was centered on the CentC cluster. These CRM1 insertions, in turn, may pave the way for the insertion of other retrotransposons that lack the ability to insert into functional centromere regions, thus further increasing the distance between the old CRM1 insertions and the present day functional centromere, which essentially consists of a CRM region flanked by CentC satellite.

Similar dynamics can be observed on centromere 2: the partial CRM1 elements at 92.5 and 92.7 Mb are older than those in or near the present-day functional centromere, from which these elements are separated by a number of more recently inserted non-CRM elements. In contrast to the centromere 5 CENH3 regions, which contain no (“R”) or only very recent CRM insertions (left half of “L”), centromere 2 contains a continuous record of CRM element insertions at its present location (Figure 2C), and therefore appears to have existed in this location for the past 3–4 million years. In contrast, centromere 5 seems to have undergone a significant lateral shift during this time period that appears to have contributed to its larger size and apparent lower CENH3 density.

### Centromere 5 has moved

By extrapolating this process, i.e. alternating CRM and non-CRM element insertions leading to changes in centromere size and location, to the more distant past for which we lack a good retrotransposon insertion record (because older insertions have been removed from the genome), the remodeling can be extended to the entire centromere 5 region as follows: the original CentC-rich centromere may have been invaded by an ancient CRM subfamily (possibly CRM4) that split the CentC cluster in two and expanded the “L” region by making it accessible to non-CRM retrotransposons that make up the bulk of “L”. As a result, the left CentC cluster (at 100.7 Mb) is no longer associated with CENH3. This was followed by insertions of CRM1 elements in the major remaining CentC cluster at the “L”/”I” border, which caused the separation of the L and R domains. This wave of CRM1 insertions may have also deleted CENH3-containing chromatin (possibly CentC), which in turn may have caused the CENH3 domain to expand into the “L” region, opening this region to CRM1 and CRM2 elements while preventing non-CRM elements from inserting. Only these most recent waves of CRM1/2 insertions can be reconstructed, as older retroelement insertions are more likely to be partially or completely removed from the genome.

The small number of retrotransposon insertions into the “R” region makes it difficult to reconstruct its history. One explanation for this dearth of CRM insertions is that the “R” region has formed relatively recently in response to the changes described above for the “L”/”I” regions. A more likely explanation may be that the lower apparent CENH3 density makes this region a less attractive target for CRM insertion than the “L” region.

Centromere 5 dramatically illustrates the centromere's ability to move locally in response to retrotransposon insertions. The left half of the current centromere 5 “L” block appears to have acquired CENH3 only during the very recent past – the density of CRM1 elements around the CentC cluster located at the “L”/”I” border suggests that prior to this the centromere was located between 103.1 and 107.4 Mb (boxes A/A' and B/B' in Figure 4). That centromere would have looked very similar to today's centromere 2, i.e. a central CentC cluster surrounded by CRM1 and CRM2 (Figure 4B). Notably the regression lines of the CRM1B/R1 elements are quite similar for centromeres 2 and 5 “L” (−2 Mb and −2.75 Mb per 1.5 million years, respectively), indicating that during the B/R1 period of activity, both centromeres 2 and 5 “L” shifted towards the short arm as a result of retrotransposon insertions at the centromere/long arm border. In the case of centromere 5 this has resulted in a gradual increase of the CRM-free region separating the “L” and “R” blocks as illustrated by the increasing distance between A/A', B/B' and C/C' (Figure 4). Although the newly formed interstitial region is relatively small (∼2 Mb), a number of fascinating questions arise from this separation, including whether the spindle binds to the “R” region, how this “pseudodicentric” chromosome is oriented and whether the two CENH3 regions of a single chromatid could bind microtubules from opposite poles, which histone variants are present in the “I” region, why there are so few CRM insertions into “R”, whether “R” would be able to function as the sole centromere of chromosome 5 and what the ultimate fate of “L” and “R” might be.

Plant genomes have the ability to purge LTR retrotransposons, and the half-life of rice retrotransposons has been estimated to be less than 6 million years [37]. The vast majority of elements available for this analysis have LTRs with κ<0.1, indicating they inserted in the past 7.7 million years. Nevertheless, this evidence shows that CRM element insertion can be followed by non-CRM insertion in the same genomic region, and vice versa. Thus it appears that centromeres, as defined by CENH3 nucleosomes, are fluid, and it is conceivable that CENH3 nucleosomes can move from adjacent sites into previously canonical chromatin. Once this occurs, CRM elements target and invade this newly formed centromere region. However, following extensive insertion of CRM elements that may initially be colonized by canonical nucleosomes, the probability of non-CRM elements inserting increases. The sum total of these interactions is illustrated by the chromosomal views of 2 and 5 (Figure 2A, Figure 3A): older CRM4 elements cluster within 30–40 Mb of the peak marking the present day functional centromere located at 90 Mb in centromere 2 and 105 Mb in centromere 5. These CRM4 elements may represent vestiges of an ancient centromere that have been pushed out of the centromere by consecutive retroelement insertions such as the ones we have documented for CRM1 elements for the past 4 million years. Alternatively, CRM4 elements may lack the centromere targeting exhibited by their cousins (CRM1,2,3) and instead preferentially target the pericentromeric heterochromatin. These CRM4 clusters are distinct from those located around 155 Mb of chromosome 2, which may be the remnant of an ancient centromere that was inactivated during the course of the corn genome consolidation following the allotetraploidization event, or alternatively, represent misassembly of this reference chromosome, which shows a break in rice/sorghum synteny in this region [2].

### CENH3 loading of CRM elements may be region-specific rather than sequence-specific

Due to the high sequence identity between elements of a particular subfamily it is difficult to determine from our pyrosequencing data whether any given recently inserted element is associated with CENH3. About half (7,330/14,598) of all CENH3 reads that had been classified as “CRM” mapped to more than one location with equal bitscores. The 18-fold enrichment of CRM1 elements in the ChIP data indicates that many CRM1 elements are associated with CENH3, but the overall 3-fold lower enrichment of CRM1 in comparison to CRM2 elements implies that the older CRM1 elements that now lie outside of the functional centromere (e.g. “I”) are indeed devoid of CENH3 nucleosomes. This is borne out by a comparison of CRM1 elements that inserted at similar times (κ = 0.026–0.035) on various regions of chromosome 5: elements that inserted within the “L” or “R” regions contain numerous perfect matches to anti-CENH3 ChIP reads, while those elements that inserted within the “I” region or on the long arm have fewer such matches (Figure S4). CENH3 loading in *Arabidopsis* has been shown to occur during the G2 phase of the cell cycle [38], while canonical nucleosomes are loaded during S phase. Like the centromeres of human and *Drosophila* [39],[40], rice [41] and corn [36] centromeres contain both CENH3 and canonical nucleosomes. CRM elements may be populated initially by canonical nucleosomes following integration. The subsequent replacement of canonical by CENH3 nucleosomes in some CRM elements may be dependent on their location relative to the center of the functional centromere, i.e. be more likely if the element has inserted into a CENH3-rich region. This could be mediated by a CENH3 loading mechanism that targets CENH3-rich regions. In other words, CRM elements appear to be associated with centromeres not because they hold an intrinsic attraction for CENH3 nucleosomes, but because they are more likely to be loaded with these nucleosomes as a result of inserting into active centromeres.

### Removal of satellite CentC sequence from centromeres 2 and 5: implications for centromere repeat succession

The high density of CRM elements in centromeres has been postulated to be conducive to intra-strand recombination between adjacent elements [10]. We suspect that such recombination between adjacent CRM elements inserted into CentC clusters will remove intervening CentC repeats, leading to the significantly reduced CentC content observed in present day B73 centromeres 2 and 5. It is noteworthy that both of these centromeres still do contain some CentC, which raises the intriguing question of whether centromeres lacking all CentC are viable, and whether a mechanism exists to restore the CentC content of CentC-depleted centromeres. Note that the CentC cluster to the left of centromere 5 block “L” is no longer associated with CENH3. CentC has successfully weathered sequential CRM invasions since the divergence from the maize/rice common ancestor 50 million years ago – yet CRM2 (and possibly the new CRM1 recombinants R4 and R5) seems to be more tightly associated with CENH3 at the present time.

### Summary

The work described here demonstrates the extraordinary value of the high quality maize genome sequence for the study of plant centromere evolution. The insights gained here could not have been provided by analysis of the smaller “model organism” genomes, rice and *Arabidopsis*, or by whole genome shotgun sequence that cannot easily be assembled in highly repetitive regions such as centromeres. The large genome of maize, which is more representative of a typical plant genome than those of the other model plants, has accumulated many relatively recent retrotransposon insertions that both shape and document its genome evolution. The fact that the maize genome has been sequenced using a minimum tiling path of all FPC contigs makes this sequence particularly amenable for repeat analysis.

In summary, we have developed a generally applicable set of methods to map and analyze centromere regions of any organism. Our approach is dependent on the availability of good genetic maps and mapping populations, identification of centromere-specific markers, a high quality genome sequence with a good physical map, anti-CENH3 ChIP followed by pyrosequencing, and FISH to support physical mapping data. The convergence of these techniques in the economically important, large-genome crop plant corn has enabled us to document the unexpected fluidity of its centromeres.

## Methods

### Repeat junction markers

Maize BAC sequences that were generated as part of the Maize Genome Project [2] and contained CentC/CRM based on BLAST homology to GenBank accessions AY321491.1 and AY129008.1 were used to develop repeat junction markers by the method of Luce et al. [24]. JunctionViewer software [30] was developed to screen the sequenced BAC reads or sequence contigs for the presence of repeats junctions between centromeric repeats (CentC, CRMs) and/or repeats from the TIGR Zea Repeats v3.0 database. The precise coordinates of the repeat junctions were determined based on BLAST homology to other *Zea mays* sequences in the high throughput genomic sequences (HTGS) database of GenBank, and primers spanning the junctions were designed manually. The junction markers were tested by PCR for polymorphism between inbreds B73 and Mo17; PCR conditions were optimized when amplification differed in intensity between the two parents. A total of 57 polymorphic markers were obtained by screening 791 repeat junction primers, and thirty-five of these were mapped using the IBM population.

### CRM2 transposon display markers

Transposon display was carried out as described [25],[26] with the following modifications. The full-length sequence of CRM2 (AY129008) was obtained from NCBI. Primers were designed to specifically amplify the flanking sequences of CRM2 but not other CRM families. Genomic DNA was digested using BfaI and PCR-amplified by pairing CRM2 primers with an adapter primer that hybridizes to the BfaI site. The primers for primary amplification were CRM2_R1 (5′- GAGGTGGTGTATCGGTTGCT) and BfaI +0 (5′- GACGATGAGTCCTGAGTAG). For selective amplification the primers were P^33^-labeled CRM2_R2 (5′- CTACAGCCTTCCAAAGACGC) and BfaI +3 selective bases (where different bases were added to the Bfa +0 primer). The final annealing temperature for selective amplification was 58°C. The PCR products were electrophoresed on 6% polyacrylamide gels, and the bands cut out for re-amplification. The re-amplified bands were either cloned and sequenced or directly sequenced from the PCR products.

### Genetic mapping

The genotypes of representative centromere repeat junction or transposon display markers were determined in 94 IBM [27] plants from a B73 x Mo17 cross. A representative centromere marker for each chromosome (Table 1) was mapped against a framework of ∼700 SSR and 700 SNP markers or the IBM population by Mike McMullen. The genetic locations from this data set were used to infer genetic position on the IBM2 2008 Neighbors map (www.maizegdb.org). Complete mapping data are available at www.maizegdb.org.

### Oat-maize addition line mapping

Sequences were mapped to chromosome 5 using an oat-maize addition (OMA) line for chromosome 5. PCR primers were designed on genic, non-genic single-copy or non-genic low-copy sequences, or on infrequently repeated sequences as long as the product was expected to be unique. Gene homologous sequences were identified by WU-BLAST (http://blast.wustl.edu) nucleotide homology alignments between BAC sequences and the Rice Annotation Project Release 5.0 Oryza genic sequences (ftp://ftp.plantbiology.msu.edu/pub/data/Eukaryotic_Projects/o_sativa/annotation_dbs/pseudomolecules/version_5.0/all.chrs/all.cds) using JunctionViewer. Repeat homology was identified by NCBI BLAST sequence alignments between target BAC or reference chromosome sequences and the HTGS database.

Two PCR reactions were performed for each primer pair – one using DNA from the B73 chromosome 5 oat-maize addition line (obtained from H.W. Rines, U. of Minnesota) and another with B73 genomic DNA as template. The annealing temperature for reactions was 60°C. Primer pairs resulting in PCR products with strong single bands were sequenced and those sequences were compared to their expected product sequence to confirm unique amplification. Of the 20 PCR primer pairs, 15 produced unique amplicons that were identical in sequence between B73 and OMA line for chromosome 5.

### Chromatin immunoprecipitation

ChIP was performed as previously described [16]. Approximately 50 g of leaf tissue harvested from seedlings of maize inbred B73 were used in ChIP using the maize anti-CENH3 antibody [15]. We obtained approximately 3 µg of immunoprecipitated DNA for pyrosequencing (GenBank Sequence Read Archive SRA009397). A small amount of the ChIPed DNA was used for FISH analysis to confirm the enrichment of the ChIPed DNA in the centromeres (Figure S3).

### Quantification of CentC and CRM sequence in reference chromosomes

CRM/CentC sequence coverage was identified by competitive WU-BLAST as described [2].

### Identification of retrotransposons and dating their insertion times

Full-length CRM elements were identified as described by Sharma and Presting [9]. Other types of retrotransposons present in the centromere 2 and 5 regions were identified using the maize retrotransposons and LTRs from the TEnest database (http://www.public.iastate.edu/~imagefpc/Subpages/te_nest.html) downloaded on 16 May 2009, as well as JunctionViewer annotations. Complete elements were identified using the reference chromosomes as a BLAST database and the complete retroelements as the query with a word size of 20 and an e-value of 1e-50. Locations within the centromere were extracted, extended to the full length of the retrotransposon plus an additional 2000 nucleotides on each side. Elements were grouped by family and aligned with the TEnest query using ClustalW [42]. LTRs and TSD were identified visually.

The TEnest LTR database and consensus LTRs of CRM were used to identify fragmented elements (due to sequence assembly errors, nested retrotransposon insertions or deletions) that could not be aligned or identified with the full-length retrotransposon. 5′ and 3′ LTRs were identified using the reference chromosomes as a BLAST database and the LTRs as the query with a word size of 20 and an e-value of 1e-50. Locations within the centromere were extracted, extended to the length of the LTR plus an additional 200 nucleotides on each side. LTRs were grouped into separate files based on subfamily and aligned with ClustalW. LTRs were sorted by location and TSDs were compared. Two LTRs of the same element type, located within 200 kb of each other and containing nearly identical TSD (i.e. the 5′ TSD of one LTR matching at least four of the five nucleotides of the 3′ TSD of the other LTR) were considered to belong to the same retrotransposon. Insertion times for these fragmented LTRs were dated based on sequence divergence using the method of San Miguel et al. [1]. Evolutionary distances (*κ*  =  estimated number of nucleotide substitutions per site) between LTR pairs with TSDs were calculated using the K2P model in MEGA version 4.0 [43]. One CRM1 retrotransposon from chromosome 5 (109.7/109.8 Mb) was dated without verifying TSDs, as this element (CRM, *κ* = 0.029) contains an insertion in its LTR – only 249 nt of its LTRs were used to calculate *κ*. Fourteen other elements with TSDs (4 CRM4, 2 CRM2 and 8 non-CRM) that contained insertions or gaps were manually truncated based on their alignment prior to estimating *κ*.

### FISH probes that distinguish CRM1 and CRM2 elements

To detect CRM1 and CRM2 subfamilies, primers specific to each subfamily were designed in the 5′ LTR, 5′ UTR-polyprotein (Plyp1) and polyprotein regions (Plyp2) (Table 1). The sequence diversity of CRM1 elements necessitated the design of multiple LTR (A, B/R1, R2, R3, R4/R5) and polyprotein (A, B) primers. CRM1 and CRM2 specific regions were amplified using *Zea mays* inbred B73 genomic DNA by 40 cycles of polymerase chain reaction (94° for 40 sec, 60° for 30 sec, and 72° for 1 min) and subsequently cloned in StrataClone PCR cloning vector pSC-A-amp-kan (GenBank accessions GQ345011-GQ345022). CRM1 and CRM2 specific FISH probe cocktails were generated by pooling their respective amplicons in equimolar amounts.

### Metaphase FISH and Fiber–FISH

Metaphase FISH was performed as described by Kato et al. [29]. Fiber-FISH procedures were performed according to Jackson et al. [44] with some modifications. For three-color detection, the biotin-labeled probe (CentC), dig-labeled probe (CRM2) and DNP-labeled probe (CRM1) were detected with far red, red and green, respectively, using three successive layers of antibodies as follows: Layer 1: rabbit anti-DNP + streptavidin 647 in TNB (0.1 M Tris-HCl pH 7.5, 0.15 M NaCl, 0.5% blocking reagent); Layer 2: biotinylated anti-streptavidin + chicken anti-rabbit 488+ mouse anti-dig in TNB; Layer 3: streptavidin 647+ rabbit anti-mouse 568 1∶200 in TNB. All antibody incubations were at 37°C; the first layer was for 1 h and the last two for 45 min each. All antibody washes were for three times of 5 min at RT using TNT (0.1 M Tris-HCl, 0.15 M NaCl, 0.05% Tween 20, pH 7.5). A final wash in PBS (0.14 M NaCl, 8 mM Na2HPO4, 1.8 mM KH2PO4, 2.7 mM KCl, pH 7.4) was performed, and the slides were drained and mounted in Vectashield without counterstain. The fluorescence signals were detected using a Hamamatsu CCD camera. The images were processed using Meta Imaging Series 7.5 software using an Olympus BX51 epifluorescence microscope equipped with FITC-Cy3-Cy5-DAPI four-way filter sets (Olympus). A conversion factor of 3 kb/µm (derived from [44],[45]) was used to approximate the physical DNA distance from the micrographs.

## Supporting Information

Figure S1 Fiber FISH map of the CentC region within B73 centromere 2. An oat-maize addition line for B73 chromosome 2 was hybridized with CentC (green) and a CRM probe (red) that does not distinguish among subfamilies CRM1, CRM2, and CRM3. The FISH images for eight different stretched fibers are shown along with the interpretation below.
(1.70 MB TIF)

Figure S2 Centromere positions on the other eight maize chromosomes. A single centromere is identified by mapped anti-CENH3 reads (top panel) on 6 chromosomes, while chromosomes 4, 6, 7, and 10 exhibit multiple ChIP peaks that are supported by centromeric repeats (bottom panel). Repeat junction and transposon display markers were used to map all functional centromere regions to the correct chromosomal location. Top panel: Moving average of 9 windows of the number of sequence reads mapped per 100 kb window using MUMmer (red line) or BLAST (purple line). Bottom panel: centromeric repeats CRM1, CRM2, CRM3, CRM4, and CentC mapped onto the reference chromosomes using competitive BLAST and graphed as number of nucleotides per 100 kb window. Centromeres 2 and 5 are shown in more detail in the text.
(1.56 MB TIF)

Figure S3 FISH of ChIPed DNA on B73 metaphase chromosomes. Note the bright centromere signal in both the nuclei and metaphase chromosomes indicating enrichment of centromeric DNA sequences.
(0.47 MB TIF)

Figure S4 CENH3 content of CRM1 elements reflects that of the surrounding genomic region. CENH3 coverage of similarly dated CRM1s (κ = 0.026–0.035) is illustrated in these computer generated JunctionViewer images. “L”, “I”, “R”, and “Long arm” denote the region where each CRM element is located. Precise reference chromosome coordinates as well as element type and κ are provided for each element. Top panel: Query sequence coverage by ChIP reads mapped at 100% identity over 100% length to a unique location (red) or any number of locations (grey) in the reference genome. Red and grey y-axis maxima are 3 and 50, respectively. Second and third panel: cross_match and BLAST homologies, respectively. Grey vertical bars indicate breaks (100 Ns) in the sequence. Blue arrows  =  CRM1 LTR, tan boxes  =  CRM polyprotein, grey  =  homology to TIGR Zea Repeats Database v3.0. Bottom panel: Red and blue arrows ≥100 nt exact match within the window. Tick marks above the elements denote 1,000 nt.
(5.07 MB TIF)

Table S1 Centromeric repeat junction markers.
(0.06 MB PDF)

Table S2 Centromeric CRM2 retrotransposon display markers.
(0.05 MB PDF)

Table S3 CENH3 and centromeric repeat density of the two chromosome arms and the centromere region of chromosome 2.
(0.04 MB PDF)

Table S4 CENH3 and centromeric repeat density of the two chromosome arms and the three distinct centromere regions of chromosome 5.
(0.04 MB PDF)

Table S5 Maximal centromeric repeat (CRM and CentC) content of any 100 kb window within centromeres 110.
(0.04 MB PDF)

Table S6 Number and type of retrotransposons identified in and near centromeres 2 and 5.
(0.05 MB PDF)

Table S7 Molecular markers used for anchoring chromosome 2 and 5 centromere regions.
(0.04 MB PDF)

Table S8 Oatmaize addition line (OMA) markers used to anchor centromere 5 BAC clones.
(0.09 MB PDF)
