# Supplementary figures and images for: Maize Centromere Structure and Evolution: Sequence Analysis of Centromeres 2 and 5 Reveals Dynamic Loci Shaped Primarily by Retrotransposons
Source: PLoS Genet. 2009 Nov 20;5(11):e1000743. doi: 10.1371/journal.pgen.1000743 (PMC2776974; doi:10.1371/journal.pgen.1000743)

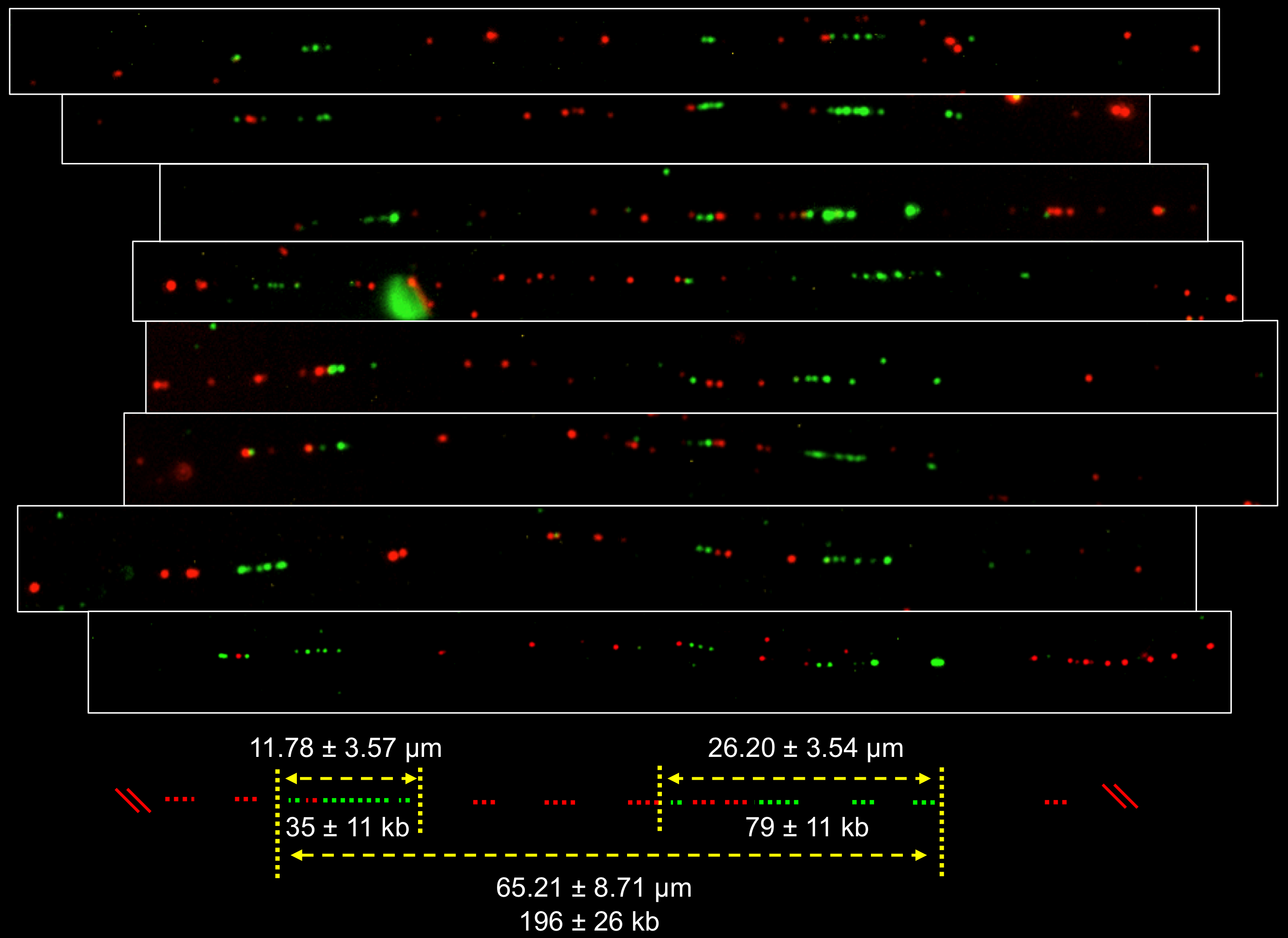

Supplement: Figure S1 — Fiber FISH map of the CentC region within B73 centromere 2. An oat-maize addition line for B73 chromosome 2 was hybridized with CentC (green) and a CRM probe (red) that does not distinguish among subfamilies CRM1, CRM2, and CRM3. The FISH images for eight different stretched fibers are shown along with the interpretation below. (1.70 MB TIF) [file pgen.1000743.s001.tif]

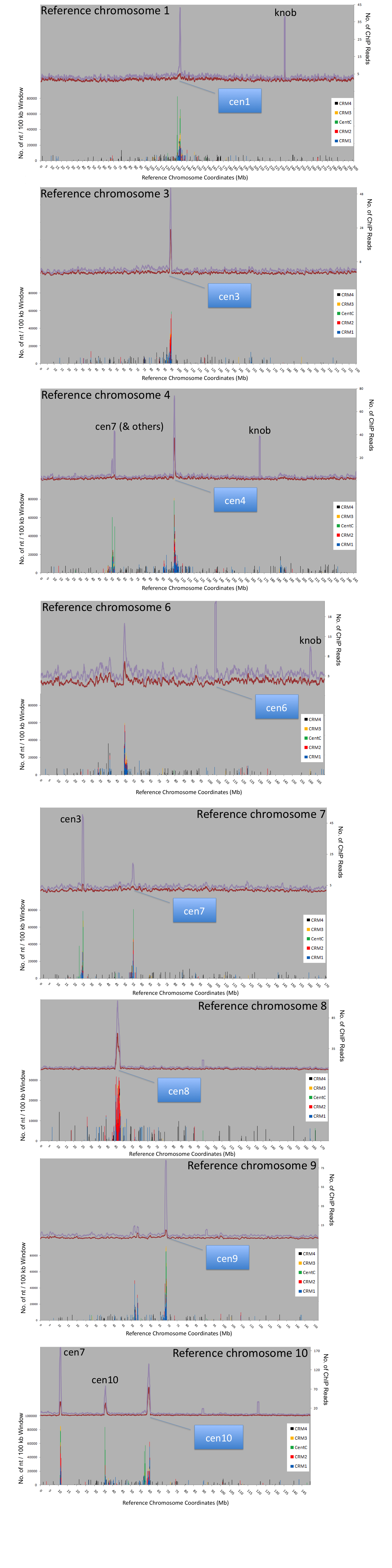

Supplement: Figure S2 — Centromere positions on the other eight maize chromosomes. A single centromere is identified by mapped anti-CENH3 reads (top panel) on 6 chromosomes, while chromosomes 4, 6, 7, and 10 exhibit multiple ChIP peaks that are supported by centromeric repeats (bottom panel). Repeat junction and transposon display markers were used to map all functional centromere regions to the correct chromosomal location. Top panel: Moving average of 9 windows of the number of sequence reads mapped per 100 kb window using MUMmer (red line) or BLAST (purple line). Bottom panel: centromeric repeats CRM1, CRM2, CRM3, CRM4, and CentC mapped onto the reference chromosomes using competitive BLAST and graphed as number of nucleotides per 100 kb window. Centromeres 2 and 5 are shown in more detail in the text. (1.56 MB TIF) [file pgen.1000743.s002.tif]

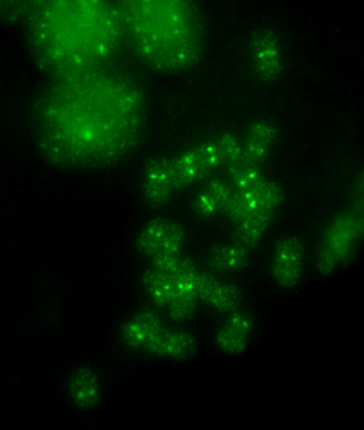

Supplement: Figure S3 — FISH of ChIPed DNA on B73 metaphase chromosomes. Note the bright centromere signal in both the nuclei and metaphase chromosomes indicating enrichment of centromeric DNA sequences. (0.47 MB TIF) [file pgen.1000743.s003.tif]

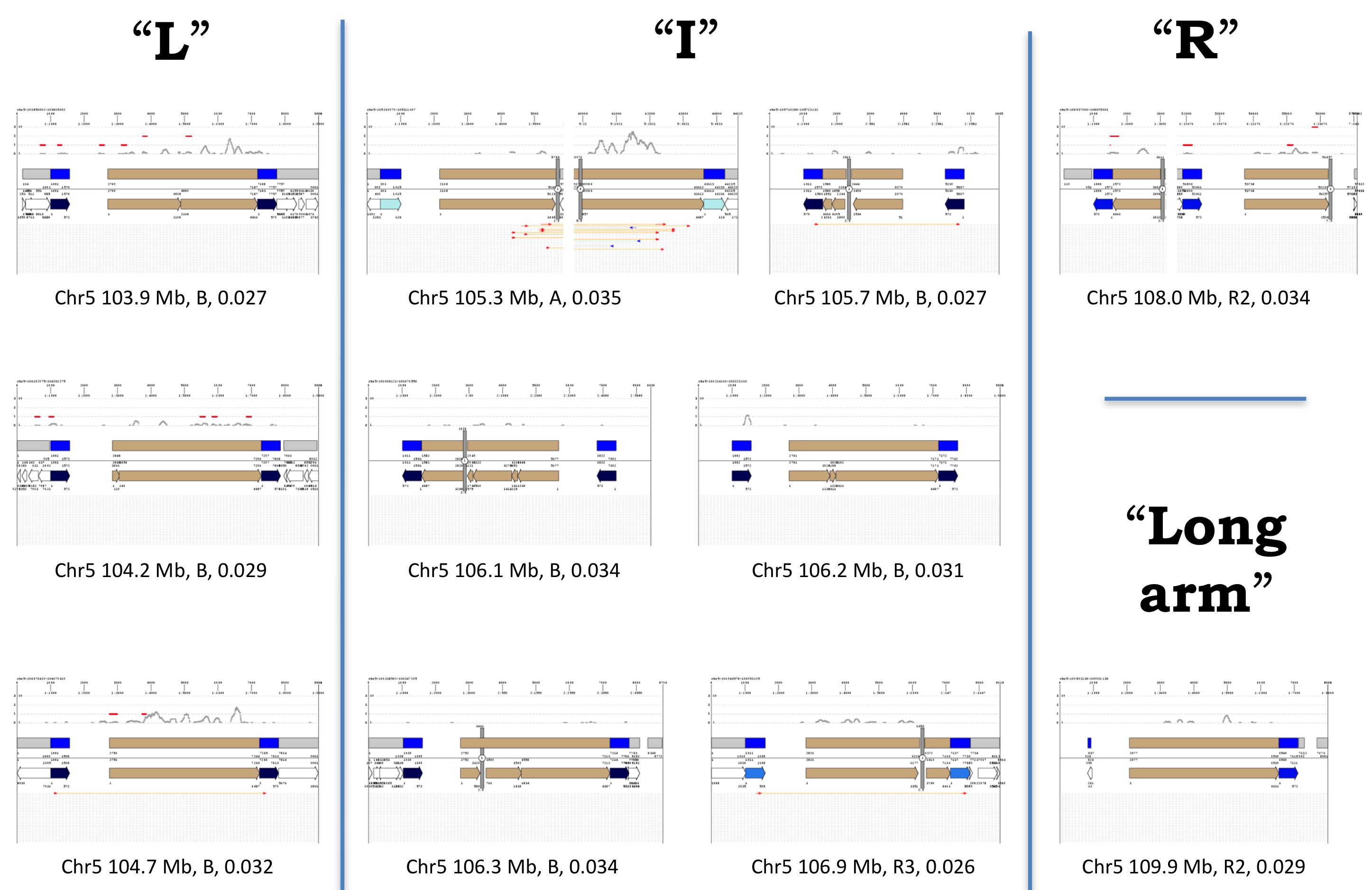

Supplement: Figure S4 — CENH3 content of CRM1 elements reflects that of the surrounding genomic region. CENH3 coverage of similarly dated CRM1s (κ = 0.026–0.035) is illustrated in these computer generated JunctionViewer images. “L”, “I”, “R”, and “Long arm” denote the region where each CRM element is located. Precise reference chromosome coordinates as well as element type and κ are provided for each element. Top panel: Query sequence coverage by ChIP reads mapped at 100% identity over 100% length to a unique location (red) or any number of locations (grey) in the reference genome. Red and grey y-axis maxima are 3 and 50, respectively. Second and third panel: cross_match and BLAST homologies, respectively. Grey vertical bars indicate breaks (100 Ns) in the sequence. Blue arrows = CRM1 LTR, tan boxes = CRM polyprotein, grey = homology to TIGR Zea Repeats Database v3.0. Bottom panel: Red and blue arrows ≥100 nt exact match within the window. Tick marks above the elements denote 1,000 nt. (5.07 MB TIF) [file pgen.1000743.s004.tif]
