# Supplementary material for: Maize Centromere Structure and Evolution: Sequence Analysis of Centromeres 2 and 5 Reveals Dynamic Loci Shaped Primarily by Retrotransposons
Source: PLoS Genet. 2009 Nov 20;5(11):e1000743. doi: 10.1371/journal.pgen.1000743 (PMC2776974; doi:10.1371/journal.pgen.1000743)
Supplement: Table S1 — Centromeric repeat junction markers. (0.06 MB PDF) [file pgen.1000743.s005.pdf]

Table S1. <u>Centromeric Repeat Junction Markers</u>. Markers are listed with the BACs from which they were derived, the two primer sequences used for amplification, amplicon size (in nt), marker type (P/A=presence/absence, SNP=single nucleotide polymorphism). The centromere to which each marker was mapped using the IBM mapping population is indicated by the marker name. Primer sequence in **bold** was used to sequence the SNP markers.

Marker Name	Centromere	BAC Name (ZMMBB)	F Primer Sequence	R Primer Sequence	Amplicon Size (nt)	Type
CenUH_01.1	1	b0010G12	GGAACCATTTCGTCGTTTTT	GTGGATGTCTTCATCATGTGG	582	P/A
CenUH_01.2	1	b0395M04	TGTCCGCATCACCTAGGATA	TCGGAATTGATGAAGACATCC	1851	P/A
CenUH_02.1	2	b0061K01	AATCTCCGCCATTCATCAAC	GATGCAGTGTCTGGAAATGG	829	P/A
CenUH_02.2	2	b0174C04	TAGTCCATCCCTGCATTTGG	GTTGATTTGTAGGGCGATGG	769	P/A
CenUH_02.3	2	b0008G14	GTTAACACACCGCCACCAA	CTCCCTGCTCCACCTCTTC	799	P/A
CenUH_02.4	2	c0149I02	GATTTTAGAAGAAAGTGCAAT	GGGACGCTTCACCCTACCATG	506	P/A
CenUH_02.5	2	c0154H24	CGCAATTTCGTTGCTGATAGA	CATGGTCCCAATAGTCGGTAAC	166	P/A
CenUH_03.1	3	c0024O03	AGGATGTCCTCATCATTGGTTC	CAAACACCAAAACACCAAAAGA	1356	P/A
CenUH_04.1	4	c0382E13	GTGATGTCCTCATCACTTCTTTTG	**CATCCGGTCCGTCTGATAGA**	1689	SNP
CenUH_05.1	5	c0026E18	CACCAAGCACTACCGGAAT	GTCATGCTATGATTGCCACTGA	1956	P/A
CenUH_06.1	6	c0462I03	GGATGTCTTCATCAGATCTCCAA	ATGGGAAGTTTTTGTTTATTTCG	1452	P/A
CenUH_07.1	7	b0346P08	TGTCTTCATCAAAAACTGAGAACA	GGGTTCATCAAATCCCTCGT	291	P/A
CenUH_07.2	7	c0447A01	CGAAGGTGTACGGGGAAATCTA	GGTCGTCAGATCCCTGGTTACT	179	P/A
CenUH_08.1	8	b0089F07	GCCTATCAGCCTATCCCACA	TGCTCACCTTGAGTGCAAAC	578	P/A
CenUH_09.1	9	c0530C10	GATGTCCGCATCAAATATGTCA	CTGTTGGGTTTATCAGCTACCAA	816	P/A
CenUH_10.1	10	b0410L22	CCTAAGGACTCATGTAGAAGCCAA	GGGTGCACATCAATGAGCTTA	1438	P/A
