# Supplementary material for: Maize Centromere Structure and Evolution: Sequence Analysis of Centromeres 2 and 5 Reveals Dynamic Loci Shaped Primarily by Retrotransposons
Source: PLoS Genet. 2009 Nov 20;5(11):e1000743. doi: 10.1371/journal.pgen.1000743 (PMC2776974; doi:10.1371/journal.pgen.1000743)
Supplement: Table S2 — Centromeric CRM2 retrotransposon display markers. (0.05 MB PDF) [file pgen.1000743.s006.pdf]

Table S2. Centromeric CRM2 Retrotransposon Display Markers.

Name[1]	GenBank Accession	BAC homology	Size (nt)	ZmB73v1[2]
1_B_AGC204	GF099546	no hit	204	not mapped
2_B_TGC288	GF099549	CH201-137F12	288	89,307,454
2_B_AGC189	GF099557	CH201-517P4	189	89,493,043
2_B_GCA370	GF099559	CH201-517P4	370	89,493,286
2_B_AGC345	GF099548	CH201-154H24, CH201-32G21	345	89,829,383
2_B_ACC381	GF099564	CH201-154H24	381	89,956,571
2_B_GCA137	GF099565	CH201-552J10	137	90,099,795
2_B_ACC194	GF099553	CH201-320J2	194	90,238,565
2_B_TTC348	GF099562	CH201-394D7	349	90,398,487
2_B_TCG152	GF099554	CH201-363H22	152	90,495,479
2_B_TGC260	GF099555	CH201-363H22	260	90,599,109
2_B_TGC205	GF099551	ZMMBBb-597K2, CH201-530G19	205	90,741,817
2_B_TTC183	GF099560	CH201-534P5	182	91,050,633
2_B_GAC236	GF099561	CH201-534P5	236	91,092,651
2_B_TGC178	GF099550	CH201-363H22	178	not mapped
2_B_TCG138	GF099556	CH201-530G19	138	not mapped
2_B_TGC188	GF099558	CH201-527D12	188	not mapped
2_B_GCA241	GF099563	CH201-394D7	241	not mapped
3_B_ACC205	GF099568	CH201-475I18	205	95,027,347
3_B_AGC310	GF099571	ZMMBBb-173J13	310	94,880,056
3_B_ACC245	GF099572	CH201-475I18	245	95,084,182
3_B_AGC144	GF099569	ZMMBBb-464B16	144	not mapped
4_B_TGC394	GF099575	ZMMBBb-522G8	394	104,339,158
5_B_TCG278	GF099578	CH201-425C22, CH201-73O10	278	101,481,735
5_B_ACC300	GF099580	CH201-124L17	300	102,113,056
5_B_TCG181	GF099579	CH201-124L17	181	102,115,031
6_B_TCG146	GF099584	CH201-567C5, CH201-430O5	146	50,002,743
6_B_ACC241	GF099582	ZMMBBb-597K2, CH201-530G19	241	not mapped
8_B_TGC202	GF099590	CH201-118G24	202	46,572,507
8_B_TGC354	GF099591	CH201-104L24	354	46,739,414
8_B_ACC181	GF099585	CH201-104L24, ZMMBBb-472G24	181	46,815,061
8_B_ACC165	GF099594	no hit	165	47,014,890
8_B_ACC361	GF099593	CH201-161G22	361	47,463,710
8_B_TGC318	GF099588	CH201-161G22	318	47,463,754
8_B_TCG175	GF099587	CH201-161G22	175	47,531,459
8_B_ACC348	GF099592	CH201-161G22	348	47,531,459
8_B_ACC225	GF099586	CH201-161G22, ZMMBBb-76N16	225	47,619,173
8_B_AGC374	GF099589	CH201-325A22	374	not mapped
10_B_AGC240	GF099596	CH201-419B14	240	59,053,330
10_B_AGC221	GF099595	CH201-364K15	221	60,397,027

[1] Marker name consists of chromosome to which marker mapped in the IBM mapping population, inbred (B = B73), selective trinucleotide used for band amplification, and sequence length, separated by "_".
[2] Reference chromosome coordinates are indicated when marker mapped to the correct reference chromosome with the highest bitscore (BLASTN).
