# Supplementary material for: Maize Centromere Structure and Evolution: Sequence Analysis of Centromeres 2 and 5 Reveals Dynamic Loci Shaped Primarily by Retrotransposons
Source: PLoS Genet. 2009 Nov 20;5(11):e1000743. doi: 10.1371/journal.pgen.1000743 (PMC2776974; doi:10.1371/journal.pgen.1000743)
Supplement: Table S3 — CENH3 and centromeric repeat density of the two chromosome arms and the centromere region of chromosome 2. (0.04 MB PDF) [file pgen.1000743.s007.pdf]

Table S3. CENH3 and centromeric repeat density of the two chromosome arms and the centromere region of chromosome 2. CENH3 density is reported as the number of anti-CENH3 reads mapped per 100kb window using MUMmer averaged either over an entire arm or centromere region. Centromeric repeats are reported as total number of nucleotides per chromosome section.

	Short arm	Centromere	Long arm
Coordinates (Mb)	87.1-89.3	89.3-91.1	91.1-93.5
CENH3 density	2.11	62.8	2.08
CRM1	31,874	209,986	55,057
CRM2	40,773	290,413	23,078
CRM3	0	0	0
CRM4	7,272	3,924	538
CentC	0	31550	0
