# Supplementary material for: Maize Centromere Structure and Evolution: Sequence Analysis of Centromeres 2 and 5 Reveals Dynamic Loci Shaped Primarily by Retrotransposons
Source: PLoS Genet. 2009 Nov 20;5(11):e1000743. doi: 10.1371/journal.pgen.1000743 (PMC2776974; doi:10.1371/journal.pgen.1000743)
Supplement: Table S4 — CENH3 and centromeric repeat density of the two chromosome arms and the three distinct centromere regions of chromosome 5. (0.04 MB PDF) [file pgen.1000743.s008.pdf]

**Table S4. CENH3 and centromeric repeat density of the two chromosome arms and the three distinct centromere regions of chromosome 5.** CENH3 density is reported as the number of anti-CENH3 reads mapped per 100kb window using MUMmer, averaged either over an entire arm or centromere region. Centromeric repeats are reported as total number of nucleotides per chromosome section.

| | Short arm | "L" | "I" | "R" | Long arm |
|---|---|---|---|---|---|
| Coordinates (Mb) | 99.3-101.6 | 101.6-104.8 | 104.8-107.6 | 107.6-108.6 | 108.6-111.1 |
| CENH3 density | 1.89 | 38.97 | 4.79 | 31.50 | 1.78 |
| CRM1 | 7,600 | 155,697 | 314,534 | 6,377 | 6,617 |
| CRM2 | 34,271 | 219,806 | 22,717 | 0 | 5,476 |
| CRM3 | 0 | 12,268 | 29,926 | 0 | 0 |
| CRM4 | 24,151 | 15,583 | 10,207 | 6,775 | 2,685 |
| CentC | 8,313 | 0 | 81,280 | 0 | 0 |
