# Supplementary material for: Maize Centromere Structure and Evolution: Sequence Analysis of Centromeres 2 and 5 Reveals Dynamic Loci Shaped Primarily by Retrotransposons
Source: PLoS Genet. 2009 Nov 20;5(11):e1000743. doi: 10.1371/journal.pgen.1000743 (PMC2776974; doi:10.1371/journal.pgen.1000743)
Supplement: Table S5 — Maximal centromeric repeat (CRM and CentC) content of any 100 kb window within centromeres 110. (0.04 MB PDF) [file pgen.1000743.s009.pdf]

Table S5. Maximal centromeric repeat (CRM and CentC) content of any 100 kb window within centromeres 1-10.

chromosome	% of 100kb window homologous to CRM or CentC
1	82.5
2	71.1
3	58.9
4	63.0
5	68.3
6	59.6
7	80.8
8	31.8
9	91.4
10	85.3
