# Supplementary material for: Maize Centromere Structure and Evolution: Sequence Analysis of Centromeres 2 and 5 Reveals Dynamic Loci Shaped Primarily by Retrotransposons
Source: PLoS Genet. 2009 Nov 20;5(11):e1000743. doi: 10.1371/journal.pgen.1000743 (PMC2776974; doi:10.1371/journal.pgen.1000743)
Supplement: Table S6 — Number and type of retrotransposons identified in and near centromeres 2 and 5. (0.05 MB PDF) [file pgen.1000743.s010.pdf]

Table S6. Number and type of retrotransposons identified in and near centromeres 2 and 5. LTR pairs within the centromere 2 (87.1 Mb – 93.5 Mb) and centromere 5 (99.3 Mb – 111.1 Mb) regions were identified computationally (see methods) and assigned to an LTR retrotransposon class.

Element	Cent2	Cent5
13075	13	50
13268	11	1
bak1	11	19
cinful	12	27
danelle	2	6
grande	4	11
gyma	5	7
ji	8	15
opie	5	12
shadow	3	10
Other:	5	17
13370	0	2
fourf	1	0
giepium	0	1
huck	0	2
klause	0	2
machievelli	0	2
reiver	0	3
rire	1	1
tim	1	2
zeon	2	2
