# Supplementary material for: Maize Centromere Structure and Evolution: Sequence Analysis of Centromeres 2 and 5 Reveals Dynamic Loci Shaped Primarily by Retrotransposons
Source: PLoS Genet. 2009 Nov 20;5(11):e1000743. doi: 10.1371/journal.pgen.1000743 (PMC2776974; doi:10.1371/journal.pgen.1000743)
Supplement: Table S7 — Molecular markers used for anchoring chromosome 2 and 5 centromere regions. (0.04 MB PDF) [file pgen.1000743.s011.pdf]

Table S7. Molecular markers used for anchoring chromosome 2 and 5 centromere regions. For details, please see Tables S1, S2 and S8.

Centromere	Marker Type	Marker Name	Reference Chromosome Start Coordinate
2	RJ	CenUH_02.2	85392883
	TD	2_B_TGC288	89307454
	TD	2_B_AGC189	89493043
	TD	2_B_GCA370	89493286
	TD	2_B_AGC345	89829383
	RJ	CenUH_02.5	89875635
	TD	2_B_ACC381	89956571
	RJ	CenUH_02.3	90060298
	TD	2_B_GCA137	90099795
	TD	2_B_ACC194	90238565
	TD	2_B_TTC348	90398487
	TD	2_B_TCG152	90495479
	TD	2_B_TGC260	90599109
	TD	2_B_TGC205	90741817
	TD	2_B_TTC183	91050633
	TD	2_B_GAC236	91092651
	RJ	CenUH_02.1	91100242
	RJ	CenUH_02.4	96005366
5	genetic	TIDP7094	99733088
	OMA	OMA_5.01	100787067
	OMA	OMA_5.02	100803288
	OMA	OMA_5.03	100930854
	OMA	OMA_5.04	100930854
	TD	5_B_TCG278	101481735
	OMA	OMA_5.05	101806921
	OMA	OMA_5.06	102032973
	OMA	OMA_5.07	102032973
	TD	5_B_ACC300	102113056
	TD	5_B_TCG181	102115031
	OMA	OMA_5.08	103305541
	OMA	OMA_5.09	103305541
	OMA	OMA_5.10	103937408
	OMA	OMA_5.11	103993212
	RJ	CenUH_05.1	105154866
	OMA	OMA_5.12	107010368
	OMA	OMA_5.13	107023617
	OMA	OMA_5.14	107686916
	OMA	OMA_5.15	107901023
	genetic	IDP8659	110637623

OMA = oat maize addition, RJ = repeat junction, TD = transposon
