# Supplementary material for: Maize Centromere Structure and Evolution: Sequence Analysis of Centromeres 2 and 5 Reveals Dynamic Loci Shaped Primarily by Retrotransposons
Source: PLoS Genet. 2009 Nov 20;5(11):e1000743. doi: 10.1371/journal.pgen.1000743 (PMC2776974; doi:10.1371/journal.pgen.1000743)
Supplement: Table S8 — Oatmaize addition line (OMA) markers used to anchor centromere 5 BAC clones. (0.09 MB PDF) [file pgen.1000743.s012.pdf]

**Table S8. Oat-maize addition line (OMA) markers used to anchor centromere 5 BAC clones.** Mapping primers were designed on genic, non-genic single copy or non-genic low copy sequences. Two PCRs were performed for each primer pair, one using OMA-5 and a second using B73 genomic DNA, and amplicons were sequenced in both directions. In all cases the sequences derived from B73 and OMA-5 genomic DNA were identical. Primer c05-08-3-f-1 was replaced with primer c05-08-3-f-2 (AGGCAGATATGGCACCTGTC) for sequencing because of poor sequence quality obtained with the former.

| MarkerName | BAC Accession Number | Start | End | Product size | Primer ID | Primer sequence | Primer type |
|---|---|---|---|---|---|---|---|
| OMA_5.01 | AC207875.2 | 133787 | 134006 | 220 | c05-01-2-f-1 | CCGTAATCTGCTGCACTTCA | Non-genic single copy |
| | | | | | c05-01-2-r-1 | CGATCATCACCAGACCAATG | Non-genic single copy |
| OMA_5.02 | AC207875.2 | 95373 | 95717 | 345 | c05-01-1-f-1 | CTAGCAAGGATGGCTCTTGG | Non-genic single copy |
| | | | | | c05-01-1-r-1 | AGTGACCGAGCTCCAACTTC | Non-genic single copy |
| OMA_5.03 | AC197005.3 | 51436 | 52182 | 747 | c05-02-2-f-1 | ATTGCAAGTTCTGGGAGCTG | Genic |
| | | | | | c05-02-2-r-1 | TACCCGGATGGAATGACAAT | Non-genic single copy |
| OMA_5.04 | AC197005.3 | 51454 | 52182 | 729 | c05-02-2-f-2 | TGGACACCGATCATGACTTC | Genic |
| | | | | | c05-02-2-r-1 | TACCCGGATGGAATGACAAT | Non-genic single copy |
| OMA_5.05 | AC182837.4 | 154823 | 156318 | 1496 | c05-08-3-f-1 | GCTGTGCAGCTTTTAGCACA | Non-genic low copy |
| | | | | | c05-08-3-r-1 | TTGGATGAAATTCACCGTCA | Non-genic low copy |
| OMA_5.06 | AC196438.3 | 95667 | 96207 | 541 | c05-10-2-f-1 | GTTTCCCTGGTGCATACGAC | Genic |
| | | | | | c05-10-2-r-1 | CAAGCAATTGAAGGACACGA | Non-genic single copy |
| OMA_5.07 | AC196438.3 | 95667 | 96248 | 582 | c05-10-2-f-1 | GTTTCCCTGGTGCATACGAC | Genic |
| | | | | | c05-10-2-r-2 | CGCCGTCTTCACTTTTAGGA | Non-genic single copy |
| OMA_5.08 | AC196144.3 | 95168 | 96044 | 877 | c05-21-2-f-1 | CTCGTCCGTGGAAAAATCC | Non-genic single copy |
| | | | | | c05-21-2-r-1 | GAGGGAGGGAGAGAAACAGC | Genic |
| OMA_5.09 | AC196144.3 | 95387 | 96044 | 658 | c05-21-2-f-2 | CGTTCCCAATTTCAGCAGTC | Non-genic single copy |
| | | | | | c05-21-2-r-1 | GAGGGAGGGAGAGAAACAGC | Genic |
| OMA_5.10 | AC182108.3 | 122183 | 122511 | 329 | c05-24-2-f-1 | TCGTGTCCAAGTCTCATTGG | Non-genic single copy |
| | | | | | c05-24-2-r-1 | TGGATACGTCACATGCTTTCT | Non-genic single copy |

**Table S8 - continued**

| MarkerName | BAC Accession Number | Start | End | Product size | Primer ID | Primer sequence | Primer type |
|---|---|---|---|---|---|---|---|
| OMA_5.11 | AC182108.3 | 43393 | 43565 | 173 | c05-24-3-f-1 | GCATCATTAGAAGCGGAAGC | Non-genic single copy |
| | | | | | c05-24-3-r-1 | CACTTTCCGTCCATTCTGCT | Non-genic single copy |
| OMA_5.12 | AC216805.3 | 20707 | 20107 | 601 | Chr5R_1_F | TAGCATTTCTGCCCTGCTTT | Genic |
| | | | | | Chr5R_1_R | TGACAAATCCTAATGGTGGGATA | Genic |
| OMA_5.13 | AC216805.3 | 7458 | 6489 | 970 | Chr5R_2_F | ACAGTGGTTGGTCCTCCAAG | Genic |
| | | | | | Chr5R_2_R | ACCCTCCAATCAGTGCATTC | Genic |
| OMA_5.14 | AC202102.4 | 42835 | 42059 | 777 | Chr5R_3_F | CAGTAAGCATGCACCGACAT | Genic |
| | | | | | Chr5R_3_R | ACAAATTTCCGATGGTTGGA | Genic |
| OMA_5.15 | AC217238.3 | 38995 | 38743 | 253 | Chr5R_4_F | GAATTGGTCTTAACCCGAGGA | Genic |
| | | | | | Chr5R_4_R | TCGACTTCTCCACCATCTCC | Genic |
